# Verification of Optimal Balloon Size in Balloon Pulmonary Angioplasty for Chronic Thromboembolic Pulmonary Hypertension

**DOI:** 10.1007/s00270-023-03489-9

**Published:** 2023-06-13

**Authors:** Shun Minatsuki, Masaru Hatano, Izumi Tanikawa, Kazutoshi Hirose, Akihito Saito, Hiroki Yagi, Norifumi Takeda, Issei Komuro

**Affiliations:** 1 Department of Cardiovascular Medicine, The University of Tokyo, 7-3-1, Hongo, Bunkyo-Ku, Tokyo, 113-8655 Japan; 2 Department of Medical Engineering, Graduate School of Medicine, The University of Tokyo, Tokyo, Japan

Balloon pulmonary angioplasty (BPA) improves hemodynamics by dilating organized thrombi using a balloon and is becoming a therapeutic method for the distal type of chronic thromboembolic pulmonary hypertension (CTEPH) [1]. In BPA, a smaller balloon is typically selected and dilates as many vessels as possible in the first session and a larger balloon for subsequent sessions [2] while there are no clear criteria for determining balloon size.

A 46-year-old man was referred to our hospital for treatment of CTEPH with dyspnea for six months. His mean pulmonary artery pressure (mPAP) was 43 mmHg, indicating distal type of CTEPH based on pulmonary angiography. BPA was selected as the therapeutic method after administration of vasodilator. Optical coherence tomography (OCT) can reveal the precise morphology of organized thrombi [3], and we used OCT to evaluate the efficacy of balloon dilatation. In the first session of the right lung, we observed web lesion of the posterior ascending branch (Fig. 1A, Supplemental Video 1). The vessel diameter (VD) was 3.0 × 2.9 mm (Fig. 1B) which did not change the reference VD of the proximal site (3.4 × 2.9 mm), and we dilated with a 3.0 mm balloon. After dilatation, the ratio of wire lumen area to vessel area (RWV) was enlarged (from 0.41 to 0.49) (Fig. 1C). He underwent another BPA session to the left lung. In the second session to the right lung six weeks later, his mPAP improved to 23 mmHg. The VD of lesion and reference were almost unchanged (lesion: 3.1 × 2.5 mm, Fig. 1D, reference: 3.2 × 2.3 mm), however, RWV was increased (0.65). To increase blood flow, we dilated with a larger-sized balloon of 4.0 mm; VD after dilatation did not change (3.0 × 2.7 mm), while RWV was increased (from 0.65 to 0.88) and the organized thrombus was tightly compressed (Fig. 1E). This indicates that the organized thrombus could be expanded without vessel over-expansion, and the blood flow improved with visible venous return (Supplemental Video 2). Serial OCT findings were summarized in Table 1. An additional BPA session to the left lung vessels was performed. The mPAP measured after four sessions, two sessions on each side of the lung, improved to 18 mmHg. His clinical and hemodynamic parameters were shown in Table 2.

**Fig. 1:**
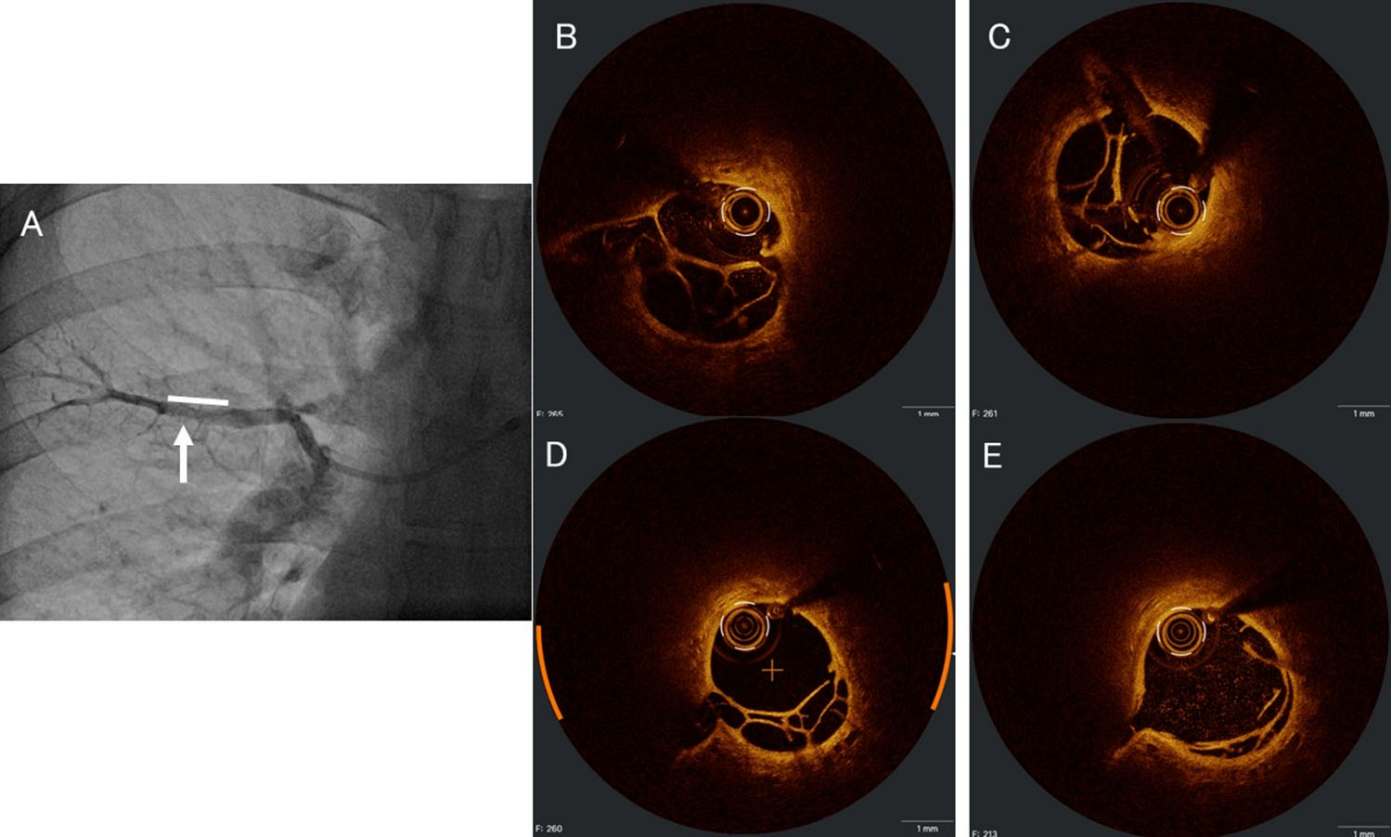
Angiogram and OCT images at the first and second BPA. **A** Angiogram before BPA (line: lesion, arrow: OCT site). **B**–**E** OCT images. **B** Pre-dilatation **C** Post-dilatation with 3.0 mm balloon **D** Pre-dilatation **E** post-dilation with 4.0 mm balloon

**Table 1:** OCT findings at the first and second BPA session to the right lung

	Dilatation with 3.0 mm		Dilatation with 4.0 mm	
	Pre	Post	pre	post
Vessel diameter (mm)	3.0*2.9	2.9*2.8	3.1*2.5	3.0*2.7
Vessel area (mm^2^)	7.1	6.7	6.0	6.5
Proximal reference diameter (mm)	3.4*2.9	3.3*2.8	3.2*2.3	3.2*2.5
Vessel diameter of wire cross lumen (mm)	2.3*1.5	2.3*1.7	2.2*2.1	2.7*2.7
Vessel area of wire cross lumen (mm^2^)	2.9	3.3	3.9	5.7
RWV	0.41	0.49	0.65	0.88

BPA: balloon pulmonary angioplasty, OCT: optical coherence tomography, RWV: ratio of wire lumen area to vessel area

**Table 2:** Clinical and hemodynamic parameters during BPA sessions

	Initial	2 months after 4 BPA session
WHO functional class	II	I
Brain natriuretic peptide (pg/ml)	197.5	18.3
Mean pulmonary artery pressure (mmHg)	43	18
Pulmonary vascular resistance (dyne/sec/cm^5^)	667	155
Six-minute walk distance (m)	628	684
Vasodilator	None	Riociguat 4.5 mg/day

BPA: balloon pulmonary angioplasty

In BPA, selecting the appropriate balloon size is crucial, as using a balloon that is too small can reduce treatment efficacy while using one that is too large can lead to pulmonary hemorrhage due to over-expansion [4]. While there is a suggestion using the diameter of the proximal site as a reference for balloon size selection [5], there is no standardized approach. The organized thrombi in CTEPH are usually stiff such as fibrous webs, dilation with a balloon approximately 1 mm oversize than the proximal reference diameter might be more effective in compressing organized thrombus and increasing blood flow, especially in cases with relatively stable hemodynamics (e.g., mPAP < 30 mmHg). To more effectively improve hemodynamics with fewer BPA sessions, further research is needed to determine the optimal approach for selecting balloon size.

## Supplementary Information

Below is the link to the electronic supplementary material.

Initial angiogram of target vessel (MP4 1031 kb)

Angiogram of target vessel after 4.0mm balloon dilatation (MP4 445 kb)
